# Differential Retinal Ganglion Cell Vulnerability, A Critical Clue for the Identification of Neuroprotective Genes in Glaucoma

**DOI:** 10.3389/fopht.2022.905352

**Published:** 2022-05-31

**Authors:** Dwarkesh Amin, Takaaki Kuwajima

**Affiliations:** Department of Ophthalmology, The Louis J. Fox Center for Vision Restoration, The University of Pittsburgh School of Medicine, Pittsburgh, PA, United States

**Keywords:** glaucoma, optic nerve, cell death, vulnerability, transcriptome, retinal ganglion cells

## Abstract

Retinal ganglion cells (RGCs) are the neurons in the retina which directly project to the brain and transmit visual information along the optic nerve. Glaucoma, one of the leading causes of blindness, is characterized by elevated intraocular pressure (IOP) and degeneration of the optic nerve, which is followed by RGC death. Currently, there are no clinical therapeutic drugs or molecular interventions that prevent RGC death outside of IOP reduction. In order to overcome these major barriers, an increased number of studies have utilized the following combined analytical methods: well-established rodent models of glaucoma including optic nerve injury models and transcriptomic gene expression profiling, resulting in the successful identification of molecules and signaling pathways relevant to RGC protection. In this review, we present a comprehensive overview of pathological features in a variety of animal models of glaucoma and top differentially expressed genes (DEGs) depending on disease progression, RGC subtypes, retinal regions or animal species. By comparing top DEGs among those different transcriptome profiles, we discuss whether commonly listed DEGs could be defined as potential novel therapeutic targets in glaucoma, which will facilitate development of future therapeutic neuroprotective strategies for treatments of human patients in glaucoma.

## Introduction

Glaucoma is a retinal neurodegenerative disease that affects 64 million people worldwide. It is estimated that the number of glaucoma patients will increase in the future (1, 2). There are several types of glaucoma: primary glaucoma including open-angle glaucoma and angle-closure glaucoma, congenital glaucoma, and secondary glaucoma. Increased IOP levels in glaucoma patients can lead to optic nerve damage and RGC loss. However, patients with a form of primary open-angle glaucoma, low tension glaucoma, also develop optic nerve damage despite having normal IOP. Importantly, the common pathological change in all types of glaucoma is loss of axonal integrity and RGC death. Unfortunately, there are no FDA-approved drugs that prevent RGC death by acting directly on RGCs themselves. Identifying therapeutic targets that directly prevent RGC death and/or enhance the capacity of RGC regrowth after axonal insult is one of the crucial steps in developing treatments for all types of glaucoma.

Using a variety of rodent models of glaucoma, a number of studies have identified intrinsic and extrinsic molecules and signaling pathways that mediate RGC survival and death (3–17). Therapeutic interventions targeting these identified genes and molecular pathways could be promising therapies. However, whether effective neuroprotection in human glaucoma patients can be achieved by targeting the same molecular pathways studied in animal disease models remains unclear. Thus, comprehensive analyses to identify beneficial molecular targets which can be commonly effective in different animal models could help to establish therapeutic molecular interventions for glaucoma patients.

In this review, first we summarize existing rodent models of glaucoma and optic nerve degeneration. We then explore the powerful impact that transcriptomic technology has made on identifying new therapeutic targets. Several transcriptomic studies with RNA sequencing (RNA-seq) or microarray have uncovered differentially expressed genes (DEGs) in RGCs or whole retina depending on disease progression, retinal regions, animal species or RGC subtypes. Among them, we select nine studies in which the most highly upregulated and downregulated genes have been identified using their own statistical criteria such as FDR *P*-value <0.05 or *P*-value <0.05 compared to control conditions and also listed in the main or supplemental figures in each study. We exclude other studies which are redundant to the selected studies due to the use of same disease models. By manually comparing gene names among those top DEGs, we identify which DEGs are commonly listed across different transcriptome profiles. We then provide the summary of previously validated functions of those common DEGs in RGCs or other cells and their molecular mechanisms. Finally, we discuss whether DEGs identified in the rodent models of glaucoma could be applied for treatments of glaucoma patients.

## Inherited and IOP-Induced Experimental Rodent Models of Glaucoma and Optic Nerve Degeneration

Genetically or spontaneously generated rodent models of glaucoma are a valuable resource for studying the genetic etiology of glaucoma and identifying future therapeutic treatments in human patients. The DBA/2J mouse strain is one of the most broadly utilized inbred mouse strains in studying glaucoma (18). DBA/2J mice begin displaying pigment dispersion, iris transillumination, iris stromal atrophy, posterior synechiae, and IOP elevation around 6 months of age. By 11 months, optic nerve atrophy, optic nerve cupping, and loss of optic nerve (60 - 90% loss) and RGCs (60 - 70% loss) are evident (16, 18–21). Genetic analysis in DBA/2J mice revealed that the iris pigment dispersion phenotype is caused by a premature stop codon mutation in the *Gpnmb* (*Gpnmb^R150X^*) gene which encodes a transmembrane glycoprotein GPNMB. Iris stromal atrophy is caused by a mutation in the b allele of *Tyrp* gene (*Tyrp1^b^*), which encodes a melanosomal enzyme, tyrosinase related protein-1, TRP-1 (22, 23). DBA/2J-*Gpnmb*^+^ mice, which are homozygous for a wild-type allele of *Gpnmb* on a DBA/2J genetic background, do not develop elevated IOP and show no glaucomatous neurodegeneration with age (24). Another inbred mouse strain, YBR/EiJ (YBR) has been characterized as a form of pigmentary glaucoma: high IOP levels are observed at six to seven months of age, and about 60% of eyes display severe axon loss with extensive gliosis and optic nerve damage by 14 months of age (25). A transgenic mouse model of glaucoma, Tg-MYOCY437H, carrying a mutation of human *MYOC* (Y437H) mimics the pathophysiology of human *MYOC*-associated glaucoma (26–28). The Tg-MYOCY437H mouse line begins to exhibit elevation of IOP, RGC loss and axon degeneration from three to five months of age, resulting in 17.6% of RGC loss by three to five months of age and 30% of RGC loss by 12 to 14 months of age (29).

Several IOP-induced experimental models have been developed, providing the advantage of acute IOP elevation and faster neurodegeneration compared to inherited rodent models. There are four major IOP-induced experimental models: laser photocoagulation, injection of microbeads, injection of silicon oil into the anterior chamber or injection of hypertonic saline through episcleral veins. (i) Laser photocoagulation to the single or multiple locations in the anterior chamber commonly causes elevation of IOP within 7 – 10 days after injury and sustained for at least a few weeks (30–35). Laser photocoagulation of both limbal and episcleral veins induces 78% of axon loss four - five weeks after injury (33, 34). Similarly, concurrent laser treatments to trabecular meshwork and episcleral veins lead to 60% of axon loss six weeks after injury (30). However, laser treatments to trabecular meshwork alone cause only 20% of axon loss six weeks after injury (30) and only 41% of axon loss as late as 24 weeks after injury (31). Laser photocoagulation of two locations such as both limbal and episcleral veins or both trabecular meshwork and episcleral veins induce around 30% of RGC loss four - five weeks after injury (33, 34) and 60% of RGC loss nine weeks after injury (30). (ii) Injection of polystyrene microbeads to anterior chamber increases IOP levels within a week, but the magnitude of IOP elevation or persistence of high IOP levels varies depending on injection volume (36), size of microbeads (37) and animal strains (38). Extended elevations of IOP for two to four weeks after injections induce 20 - 40% of axon loss and 25 – 38% of RGC loss (36, 37, 39). (iii) Clinical reports that intravitreal injections of silicone oil, which is normally used to treat complex retinal detachments, can lead to high IOP elevation and post-operative secondary glaucoma in humans (40–42). This evidence supports the development of the silicone oil-based rodent model of glaucoma. Direct injections of silicone oil into the anterior chamber leads to rapid IOP elevation within a few days, and the levels remain high for eight weeks. 88% of peripheral RGC loss and 65% of axon loss are appreciated eight weeks after injections (43). (iv) Injections of hypertonic saline through episcleral veins lead to sclerosis of trabecular meshwork and anterior chamber angle, resulting into chronic and constant IOP elevation for a few weeks to months (44–47). Rodents with more than 70% of RGC loss clearly show progressive cupping and abnormal ERG results (47).

The rodent intraorbital optic nerve crush model has been broadly utilized as a model of traumatic optic neuropathy to identify the cellular and molecular mechanisms underlying RGC death and survival, axon degeneration, and axon regeneration (48). Optic nerve crush and ocular hypertension show differing degenerative phenotypes, such as the magnitude of RGC subtype death and topography of RGC loss (49). However, several studies have demonstrated that the optic nerve crush model and inherited or IOP-induced experimental rodent models of glaucoma trigger RGC degeneration through mediating the same downstream molecules, such as Bax (50, 51) and Jun (52, 53). Optic nerve crush causes rapid neurodegeneration, with 20%, 47% and 66% of RGC loss and 38%, 58% and 67% of axon loss at three, five and seven days after injury, respectively (54). The earlier optic nerve transection model (55) shows slightly more severe pan-RGC death than the optic nerve crush model, but the magnitude of death of RGC subtypes is comparable between the two models (56). Establishing multiple rodent models of glaucoma and optic neuropathies enables us to clarify the pathology as observed in glaucoma patients and to find future therapeutic molecular interventions.

## Identification of Neuroprotective Genes by Comparative Transcriptome Analysis

### I. Disease Progression

**Figure 1:**
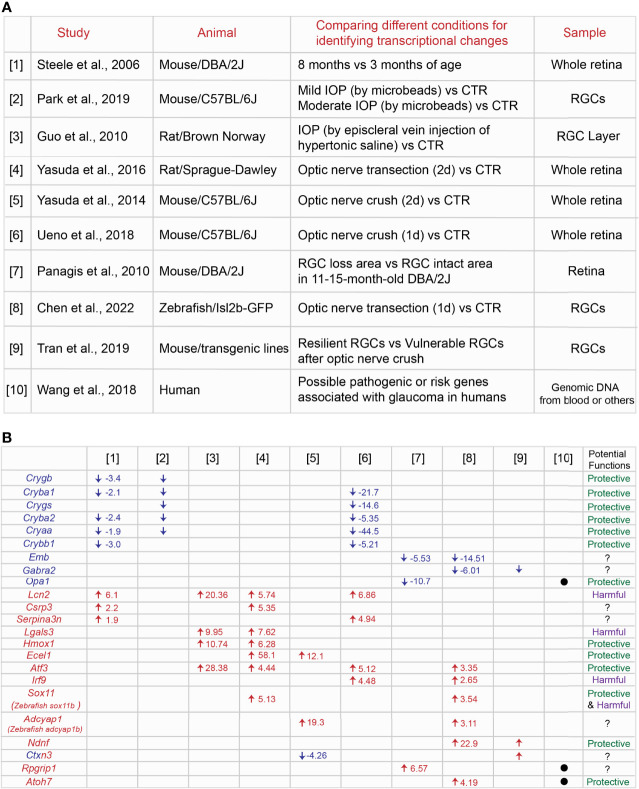
Identification of common genes among different transcriptome profiles at the specific conditions in the animal models of glaucoma. **(A)** Summary of nine [1] – [9] transcriptome studies in the retina or RGCs in a variety of animal models of glaucoma and [10] possible risk genes associated with glaucoma in humans. **(B)** Common genes listed across different transcriptome profiles and the fold change of each gene under the specific condition. Potential protective or harmful effects of common DEGs on RGCs in glaucoma have been discussed in the main text.

RGC loss and axonal degeneration in glaucoma are irreversible, and the magnitude of neurodegeneration worsens over time. In a common hypothesis, the balance between decreasing expression of neuroprotective genes and/or increasing expression of cell death-induced genes could decide the final neurodegenerative outcomes. Several studies have shown RNA-seq profiles at different time points of neurodegeneration in inherited and IOP-induced experimental rodent models of glaucoma (**Figure 1A**, **Supplemental Table 1**). [1] The retina of 3 month-old (before disease onset) and 8 month-old DBA/2J mouse (after IOP elevation) (57), [2] RGCs of the eye with mild or moderate IOP elevation (mild IOP increase:1< mmHg <4; moderate IOP increase: ≥4 mmHg) by injections of polystyrene microbeads and RGCs of the normal eye (58), and [3] the retinal ganglion cell layer (RGCL) of the eye with IOP elevation by aqueous outflow obstruction following injecting hypertonic saline unilaterally into episcleral veins and RGCL of the normal eye (59), [4] the retina two days after optic nerve transection and the uninjured retina (60), [5] the retina two days after optic nerve crush and the uninjured retina (61), and [6] the retina one day after optic nerve crush and the uninjured retina (62) have been utilized for RNA-seq or microarray analysis to identify DEGs in each study. First, we compared top DEGs listed in [1] – [6]. We found that crystallin superfamily members (*Crygb*, *Cryba1, Crygs, Cryba2*, *Cryaa*, *Crybb1*), *Lcn2* (lipocalin 2), *Lgals3* (galectin-3), *Hmox1* (heme oxygenase-1), *Ecel1* (endothelin converting enzyme like 1), *Atf3* (activating transcription factor 3), *Csrp3* (cysteine and glycine rich protein 3), and *Serpina3n* (serpin family A member 3) are commonly upregulated or downregulated (**Figure 1B**). Among these common DEGs, we summarize the information of expression and functions of crystallin superfamily members (*Crygb*, *Cryba1, Crygs, Cryba2*, *Cryaa*, *Crybb1*), *Lcn2*, *Lgals3 Hmox1*, *Ecel1* and *Atf3*. We discuss whether these DEGs could be key common therapeutic targets for treatments of glaucoma.

#### *Crygb, Cryba1, Crygs, Cryba2, Cryaa, Crybb1* - Crystallin Superfamily Members

*Crygb*, *Cryba2*, *Cryaa*, *Cryba2* are commonly downregulated in degenerating RGCs or retina as seen in [1] (57), [2] (58) and [6] (62) (**Figure 1B**). Crystallins include the α, β, and γ subtypes, and the superfamily members are expressed in normal RGCs, but they are downregulated in IOP-induced experimental models of glaucoma (63, 64). Although α-crystallins are downregulated after optic nerve crush, overexpression of αA- and αB-crystallins enhance RGC survival after injury (65). Injection of α-crystallin protein into the vitreous space after optic nerve crush also attenuate optic nerve degeneration (66). Crystallins family members carry out their anti-apoptotic functions through mediating different molecular pathways. They bind to Bax and Bcl-X and repress the translocation from the cytosol into mitochondria during staurosporine-induced cell death (59). They also prevent the activation of caspase 3 and activate PI3K (67) and attenuate endoplasmic reticulum stress (68). Microglial activation and TNF-α and iNOS release are repressed by crystallins (69). Thus, crystallins could be a potential regulator for RGC protection through mediating several molecular pathways in glaucoma.

#### *Lcn2* (Lipocalin 2)

*Lcn2* is commonly upregulated in neurodegeneration as shown in [1] (57), [3] (59), [4] (60) and [6] (62) (**Figure 1B**). Lipocalin-2 (encoded by *Lcn2*) is a secreted protein that belongs to the Lipocalins, a group of transporters of small lipophilic molecules, and the expression is elevated after administration of lipopolysaccharide (LPS) (70). Several studies have demonstrated that lipocalin-2 has neurotoxic effects. Recombinant lipocalin-2 protein induces apoptosis of neurons *in vitro* (71). Lipocalin-2 levels are elevated after intracerebral hemorrhage due to the brain injury, and loss of lipocalin-2 reduces microglial activation, brain swelling, brain atrophy, and neurologic deficits compared to control (72). Similarly, thrombin-induced brain swelling, blood-brain barrier disruption, neuronal death and neurologic deficits are markedly reduced by loss of lipocalin-2 (73). After transient middle cerebral artery occlusion, lipocalin-2 expression is upregulated in astrocytes and endothelial cells, and loss of lipocalin-2 attenuates infarct volume, neurologic deficit, and inflammatory response (74). Moreover, the expression is induced in reactive astrocytes in the rodent model of ALS, leading to neuronal death (75). Most recently, in the IOP-induced neurodegeneration *in vitro* model, recombinant lipocalin-2 protein leads to increased RGC death (76). Thus, lipocalin-2 could be a neurotoxic factor expressed in non-neuronal cells so that suppression of lipocalin-2 expression or blocking the functions could prevent RGCs from death in glaucoma.

#### *Lgals3* (Galectin-3)

Another commonly upregulated gene in neurodegeneration is *Lgals3* as seen in [3] (59) and [4] (60) (**Figure 1B**). Galectin-3 (Gal3) (encoded *lgals3*) is a member of the lectin family. In the ischemic injury model, Gal3 is expressed in activated microglial cells at the lesion site. Loss of Gal3 exacerbates ischemic damage and increases neuronal apoptosis after cerebral ischemia. This is mediated by an elevation of IL-6 and SOCS3 expression (77). Moreover, treatments with recombinant Gal3 protein reduce neuronal death and brain damage and improve post-ischemic functional recovery (78). This evidence indicates that Gal3 plays critical roles as a neuroprotective factor. However, neurotoxic roles for Gal-3 by immune cells have been also reported. Administration of neutralizing antibodies against galectin-3 attenuates the expression of pro-inflammatory markers such as IL-1β and IL-6 and exerts neuroprotection in the cortex and hippocampus after head injury (79). Loss of Gal3 also enhances RGC protection and reduces the number of degenerated axons after optic nerve crush (80). Taken together, Gal-3-mediated functions may differ depending on the stage of progression of injury and glaucoma.

#### *Hmox1* (Heme Oxygenase-1)

*Hmox1* is upregulated in neurodegeneration in [3] (59) and [4] (60) (**Figure 1B**). Heme oxygenase (HO) enzymes degrade heme to form biliverdin, iron, and carbon monoxide (CO) gas. Heme oxygenase-1 (HO-1, encoded by *Hmox1*) exerts neuronal and non-neuronal cell protection after visceral organ injury (81) and brain ischemic injury (82). In the retina, overexpression of *Hmox1* adenovirus attenuates RGC loss after pressure-induced ischemia (83). Thus, Hmox1 could play a role as a critical neuroprotective factor in glaucoma. On the other hand, light-induced retinal degeneration causes photoreceptor degeneration and increases *Hmox1* expression. High expression of *Hmox1* induces photoreceptor degeneration even without light stress (84). Taken together, Hmox1 overexpression has a neuroprotective role in only RGCs, but not other retinal cells.

#### *Ecel1* (Endothelin Converting Enzyme Like 1)

*Ecel1* is upregulated after optic nerve crush as shown in [4] (60) and [5] (61) (**Figure 1B**). Endothelin-converting enzyme-like 1 (Ecel1/DINE, encoded by *Ecel1*) has been identified as an elevated gene response upon nerve injury in the rat brain (85). In the retina, knockdown of *Ecel1* by AAV2-CRISPR/CAS9 virus leads to severe RGC loss at four days after optic nerve crush (86). In contrast, loss of Ecel1 has no impacts on RGC survival/death rates at seven days, two and four weeks after optic nerve crush compared to control. Intriguingly, loss of Ecel1 enhances RGC axon regeneration only when Zymosan, a potent monocyte activator and promotes axonal regeneration is present (87). Thus, Ecel1 could exert RGC protection only at the early stages of RGC degeneration in glaucoma.

#### *Atf3* (Activating Transcription Factor 3)

*Atf3* is commonly upregulated in [3] (59), [4] (60), [6] (62), and [8] (88) (**Figure 1B**). ATF3 is a member of the basic leucine zipper (bZip) family of transcription factors, and ATF3 is highly conserved across different species: mouse and zebrafish *Atf3* orthologs share 95% and 71% identity with human *ATF3* (89). In the optic nerve crush model, *Atf3* is preferentially upregulated in αRGCs, one of the RGC subtypes resistant to the injury signals. Overexpression of *Atf3 via* AAV2 viruses (wherein ~80% of RGCs are infected) enhances RGC survival after optic nerve injury (90). The other study has demonstrated that hippocampal neurons die due to growth factor withdrawal, application of staurosporine or NMDA, or oxygen–glucose deprivation, while ATF3 inhibits neuronal death induced by these toxic factors (91). One of potential molecular pathways for RGC protection by ATF3 is through CREB binding to the *Atf3* promoter and subsequent induction of expression (91). CREB is a downstream factor of CaMKII, which shows high neuroprotective effects in NMDA- and IOP-induced RGC degeneration models and after optic nerve crush (92). Thus, CaMKII-CREB-ATF3 molecular axis could enhance RGC survival in glaucoma.

### II. Region-Specific Retinal Effects

One of the notable pathological features at the early stages of neurodegeneration in glaucoma is that retinal axon degeneration and loss of RGCs does not occur evenly in the whole retina. Both diffuse and local patterns of RGC loss (93, 94) and specific regions of retinal axon degeneration (95) are observed in glaucoma patients. The rodent models of glaucoma also recapitulate geographically diffuse and focal/regional patterns of RGC loss and axon degeneration (21, 96–102). However, little is known about whether the same molecular mechanisms mediate neurodegeneration in all retinal regions. Most recently, our study has shown that RGCs in the peripheral ventrotemporal (VT) retina are more vulnerable to degenerative signals after optic nerve crush compared to RGCs in other retinal regions. We also demonstrated that neurodegenerative signals conveyed from the optic nerve to VT RGCs after ONC are mediated by SERT-integrin β3 molecular axis, leading to an acute reduction in the expression of GPNMB, which has an RGC protective capacity. In contrast, GPNMB expression in other retinal regions decline slowly. Thus, these molecular interactions confer region-specific RGC vulnerability after optic nerve crush (103). To understand the molecular mechanisms of focal RGC loss in glaucoma, one study has shown through RNA-Seq analysis: [7] the retinal areas where RGCs have already died and the retinal areas where RGCs continue to survive in 11 - 15 month-old DBA/2J mouse (104) (**Figure 1A**, **Supplemental Table 1**). We compared top DEGs between [7] and [1] – [6], but we are unable to find any common DEGs. However, we focus on *Opa1* (OPA1 mitochondrial dynamin like GTPase) since *Opa1* is functionally related to *Hmox1* which has been discussed above. Here, we discuss whether *Opa1* could be involved in RGC protection through the mitochondrial pathway with other DEGs such as *Hmox1* (**Figure 1B**).

#### *Opa1* (OPA1 Mitochondrial Dynamin Like GTPase)

OPA1 is an inner mitochondrial membrane protein, and loss of OPA1 induces mitochondrial membrane potential reduction and fragmentation of mitochondrial network (105). *OPA1* haplo-insufficiency is responsible for the most common form of autosomal dominant optic atrophy (ADOA), a neuropathy leading to degeneration of RGCs and the optic nerve (106). The *Opa1* mutant mouse, a mouse carrying a pathogenic mutation in *Opa1* shows a significant reduction (35 - 41% decrease) of RGCs compared to WT mice and also axon degeneration with age. Electroretinography (ERG) responses are unaffected, but visually evoked potential (VEP) measurements show significantly reduced amplitudes in the mutant mouse (107, 108). One of the molecular mechanisms underlying Opa1-mediated functions is that BNIP3, a pro-apoptotic member of the Bcl-2 family is induced by stresses such as hypoxia, and it mediates mitochondrial fragmentation and apoptosis by disrupting OPA1 complex (109, 110). Moreover, *Opa1* is directly linked to glaucomatous neurodegeneration. In the IOP-induced glaucoma model by translimbal laser photocoagulation of the trabecular meshwork, overexpression of OPA1 *via* AAV2 viruses attenuates loss of RGCs through reducing expression of BAX and improving mitochondrial health and mitochondrial surface area (111). Thus, *Opa1* could maintain mitochondrial quality, leading to RGC protection in glaucoma.

#### Hmox1 - Opa1 Pathway

As described above, the heme oxygenase-1 (HO-1, encoded by *Hmox1*) is commonly identified in [3] (59) and [4] (60), and it protects neurons and non-neuronal cells after injury (81, 82). Intriguingly, since the heme oxygenase-1 (HO-1; *Hmox1*) enzyme system is important for cell protection against oxidative damage in the cardiovascular system, loss of HO-1 shows a reduction in expression of Opa1 and other factors, leading to disruption of mitochondrial quality and then cell death (112). Moreover, loss of HO-1 gene in adipocyte cells reduces thermogenic mitochondrial fusion and fission genes such as *Opa1* (113). Thus, vulnerable RGCs locally located in the retina could be determined by dysfunctions of the mitochondrial pathways, whose importance has been also described by the other study (16).

### III. Animal Species

Most RGCs die in glaucoma patients and in the rodent models of glaucoma. However, unlike mammals, zebrafish possess high neuroprotective and regenerative capacity in the retina: in the optic nerve crush models, ∼75% of zebrafish RGCs survive after optic nerve injury, even up to 7-weeks post-injury (114). Zebrafish also show robust regenerative responses, regenerate RGC axons and connect to target regions in the brain (115–117). Several transcriptome studies using the retina or RGCs two days (118), three days or later after optic nerve crush (119) have identified DEGs which could potentially regulate axon regeneration. Most recently, to test the hypothesis that RGC protective signals could be acutely evoked after optic nerve damage, we performed RNA-seq analysis using zebrafish RGCs within 24 hours after optic nerve transection [8] (88) (**Figure 1A**, **Supplemental Table 1**). Among top DEGs, *stat3* and other molecules related to Jak/Stat pathways are highly upregulated after injury, and we found that Jak/Stat pathways mediate RGC protection while microglia/macrophages kill RGCs after optic nerve transection (88). To explore additional neuroprotective genes in RGCs across species, we compared transcriptome profiles between [8] zebrafish RGCs after optic nerve transection (88) and others in the rodent glaucoma and optic nerve injury models [1] – [7]. Intriguingly, zebrafish *atf3* (activating transcription factor 3), *irf9* (interferon regulatory factor 9), *sox11b* (mouse *Sox11*) (SRY-Box transcription factor 11), *adcyap1b* (mouse *adcyap1*) (adenylate cyclase activating polypeptide 1) and *emb* (embigin) are commonly upregulated or downregulated (**Figure 1B**). Among those common DEGs, since *Atf3* has been already discussed above, here we discuss whether other common DEGs such as *irf9* and *sox11* in mammals could be potential therapeutic targets in glaucoma.

#### *Irf9* (Interferon Regulatory Factor 9)

*Irf9* is upregulated in optic nerve crush models [6] (62) and [8] (88) (**Figure 1B**). Interferon regulatory factor 9 (IRF9 encoded by *Irf9*) is a transcription factor that regulates innate immune responses. Although there is no direct evidence that IRF9 is involved in RGC protection or death in glaucoma or after optic nerve injury, some studies have shown that IRF9 is connected with neurological pathology (120, 121). IRF9 expression is elevated in neurons after brain ischemia/reperfusion injury, and it potentiates neuronal death *via* suppression of Sirt1 expression and acetylation of p53 (122). Thus, *Irf9* could induce RGC death after nerve injury and in glaucoma in mammals, while in zebrafish, other neuroprotective factors may be more active to overcome IRF9-indcued degenerative signals in RGCs.

#### *Sox11 (Zebrafish Sox11b)* (SRY-Box Transcription Factor 11)

*Sox11* is upregulated in optic nerve crush models [4] (60) and [8] (88) (**Figure 1B**). *Sox11*, a transcription factor is highly expressed in developing RGCs, and it is a critical regulator for RGC differentiation and retinal axon guidance during development (123–126). Although Sox11 expression in RGCs is downregulated after postnatal stages (126), overexpression of Sox11 protects specific RGC types, but not pan-RGCs, and promotes retinal axon regeneration after optic nerve injury (127–129). One of molecular mechanisms is that overexpression of Sox11 increases expression levels of anti-apoptotic factor, *Bcl2.* However, Sox11 kills αRGCs, one of the resistant RGC subtypes and reduces expression of *Spp1*, a marker for αRGCs after optic nerve injury (127, 129). Thus, Sox11 could be a unique and selective neuroprotective factor in glaucoma.

### IV. RGC Subtypes

46 RGC types in adult mouse retina display differential RGC vulnerability and show distinct molecular signatures, some of which have been identified as novel neuroprotective and/or axon regeneration-related genes using single RNA-seq analysis by [9] (130) (**Figure 1A**, **Supplemental Table 1**). For instance, αRGCs are resistant to injury signals after optic nerve crush (131). Moreover, *Timp2* and *Prph* are highly expressed in resilient RGC types, and overexpression of these factors induces RGC protection after optic nerve injury. In contrast, *Crhbp* and *Mmp9* are highly expressed in vulnerable RGCs, and loss of these factors enhances RGC survival (130). Thus, clarifying molecular signatures in resilient RGC subtypes compared to vulnerable RGC subtypes after optic nerve injury enable us to identify them as novel neuroprotective factors in glaucoma. Thus, we compared transcriptome profiles between [9] (130) and [1] – [8]. We found that *Ndnf* (neuron derived neurotrophic factor), *Ctxn3* (cortexin 3) and *Gabra2* (gamma-aminobutyric acid type A receptor subunit alpha2) are commonly regulated (**Figure 1B**). Among them, we focus on *Ndnf* and discuss potential functions in RGCs.

#### *Ndnf* (Neuron Derived Neurotrophic Factor)

*Ndnf* is upregulated in injured zebrafish RGCs [8] (88) and highly expressed in resilient RGCs [9] (130) after optic nerve injury (**Figure 1B**). Neuron-derived neurotrophic factor (NDNF encoded by *Ndnf*) is a glycosylated, disulfide-bonded protein. In the retina, NDNF is more highly expressed in resilient RGCs compared to vulnerable RGCs, and overexpression of *Ndnf via* AAV2 virus in RGCs promotes RGC survival after optic nerve injury (130). NDNF also has the neuroprotective effects in other CNS neurons. In the hippocampus neuron cultures, NDNF supports neuronal survival and promotes neurite growth and neuron migration (132). Although the molecular mechanisms underlying NDNF-mediated neuronal protection are still unclear, NDNF could be identified as a potential therapeutic gene in glaucoma.

## Potential Therapeutic Targets for Glaucoma Patients

A large number of studies on glaucoma inheritance in humans have identified loci that could be associated with specific glaucomatous phenotypes or genetic mutations, suggesting that genetic factors could trigger the initiation of glaucoma. The review article by Wang et al. (133) show that 22 loci of glaucoma are listed and includes the relevant genes such as myocilin (MYOC), optineurin (OPTN), cytochrome P450 subfamily I polypeptide 1 (CYP1B1) and others. [10] These authors also display 74 possible pathogenic or risk genes associated with glaucoma (133) (**Figure 1A**, **Supplemental Table 1**). Moreover, single nucleotide polymorphisms (SNPs) and larger variations including copy number variations could also impact the progression and magnitude of glaucomatous phenotypes. Thus, we compare top DEGs identified in [1] – [9] with 74 possible pathogenic or risk genes listed in [10]. We found that *OPA1* (OPA1 mitochondrial dynamin like GTPase), *RPGRIP1* (RPGR Interacting Protein 1) and *ATOH7* (Atonal BHLH Transcription Factor 7) are commonly listed (**Figure 1B**). Indeed, these genes have been associated with glaucoma in humans: *OPA1* (134, 135), *RPGRIP1* (136) and *ATOH7* (137). OPA1 in rodent RGCs has been discussed above, and it could be a potential neuroprotective gene. Expression and functions of ATOH7 in developing RGCs have been investigated, with ATOH7 mediating RGC survival during development (138). Moreover, most recently, 127 loci associated with open-angle glaucoma have been identified, with specific genes, such as *RERE*, *VCAM1*, *ZNF638*, *SMAD6* potentially conferring open-angle glaucoma risk (139). Among those risk genes, *MAPT* is also listed as one of DEGs in the IOP-induced glaucoma model [3] (59). The microtubule-binding protein tau (tau encoded by *MAPT*) enhances RGC death after optic nerve crush (140). Alternative splicing of *MAPT* produces distinct tau isoforms which induce RGC death in the rodent model of Alzheimer’s disease (141). Thus, MAPT could be a mediator of RGC death in glaucoma.

## Concluding Marks

Combined analytical studies using well-established animal models of glaucoma and transcriptomic gene expression profiles have provided critical molecular insights into how RGCs can be preserved in glaucoma. In this review, we performed comparative analyses of several transcriptomic profiles using different glaucoma models. We found that some of the DEGs are commonly listed across different species and/or animal disease models, suggesting that they are most likely to be effective therapeutic targets for treatments of RGC degeneration in glaucoma. Because these DEGs have been identified from different cellular sources, such as single RGCs, RGC layer or whole retina, they could affect RGC survival or death through cell autonomous or non-cell autonomous mechanisms. Even if functional studies of these common DEGs unveil no role for them in neurodegeneration, they can still be utilized as molecular markers to inform us about the magnitude of RGC degeneration and/or animal- and cell-type specific degenerative outcomes. There are many other DEGs that we were unable to cover in the scope of this review but may ultimately play important roles in neuroprotection. Several key review articles have previously organized and described relevant genes and molecular mechanisms of neurodegeneration in glaucoma (142–144). Most recently, Wang et al. (145) have also summarized commonly upregulated or downregulated genes by comparing transcriptomic data in several glaucoma mouse models and optic nerve crush models, most of which we have also included in this review. The deep molecular insights into neurodegeneration and neuroprotection, in particular in immune-mediated and other molecular pathways, provided by Wang et al. (145), complement the focus of our review. Together, these primary experimental studies and further cross-analysis through review articles, including ours, will lead to a better understanding of common and differing molecular mechanisms that underly neurodegeneration in disease and injury conditions. Further studies using the existing or novel rodent models of glaucoma with current and new transcriptomic gene expression profiles under a certain condition will be beneficial for gaining more knowledge of targeted molecules for future gene therapy. Moreover, in the comparison of DEGs identified in transcriptomic profiles to disease susceptibility loci identified in epidemiological datasets, only a few DEGs are matched to glaucoma risk alleles in humans. Indeed, many susceptibility loci are related to regulation of IOP since they are expressed in the trabecular meshwork or angle tissues. Thus, further studies aiming at identification of risk genes directly connected to neurodegeneration in glaucoma patients will be needed.

There are still many unanswered questions: how many genes or interventions are required for completely preventing or delaying neurodegeneration? Can we use the same molecular interventions in all types or specific types of glaucoma? What visual functions can be maintained by one or the other gene manipulations in all or specific RGC subtypes? What evaluation system (e.g. 3-D human retinal organoid) is the most reliable to test the neuroprotective efficacy of candidate genes in human glaucoma RGCs? Further research will give answers to these critical questions that are key to development and proper choice of neuroprotective interventions that take into account disease stage and the types of glaucoma.

## Author Contributions

All authors contributed to the article and approved the submitted version.

## Funding

This work was supported by fund from Research to Prevent Blindness/Ernest & Elizabeth Althouse/Dolly Green Special Scholar Award and Startup funds from the Department of Ophthalmology University of Pittsburgh School of Medicine and the Eye and Ear Foundation of Pittsburgh.

## Conflict of Interest

The authors declare that the research was conducted in the absence of any commercial or financial relationships that could be construed as a potential conflict of interest.

## Publisher’s Note

All claims expressed in this article are solely those of the authors and do not necessarily represent those of their affiliated organizations, or those of the publisher, the editors and the reviewers. Any product that may be evaluated in this article, or claim that may be made by its manufacturer, is not guaranteed or endorsed by the publisher.

## Supplementary Material

Supplementary Table 1 Top differentially expressed genes in nine transcriptome studies in the retina or RGCs in different animal models of glaucoma and a list of possible risk genes for glaucoma in humans.
